# Synergistic Antimicrobial and Antiviral Efficacy of Chitosan–Silver Nanocomposites Against Major Pathogens of *Bombyx mori*: In Vitro and In Vivo Evaluations

**DOI:** 10.3390/insects17040403

**Published:** 2026-04-08

**Authors:** Tao Xu, Zi Liang, Xinhao Jiao, Lulai Wang, Haoran Zhong, Ping Wu

**Affiliations:** 1 School of Biotechnology, Jiangsu University of Science and Technology, Zhenjiang 212100, China; xu839597748961213@163.com (T.X.); 15850025782@163.com (Z.L.); 19503840347@163.com (X.J.); wanglulai22@163.com (L.W.); 2 Shanghai Veterinary Research Institute, Chinese Academy of Agricultural Sciences, Shanghai 200241, China; haoranzhong@shvri.ac.cn; 3 Key Laboratory of Silkworm and Mulberry Genetic Improvement, Ministry of Agriculture and Rural Affairs, Sericultural Scientific Research Center, Chinese Academy of Agricultural Sciences, Zhenjiang 212100, China

**Keywords:** *Bombyx mori*, silkworm pathogens, chitosan–silver nanoparticles, *Serratia marcescens*, minimum inhibitory concentration, oxidative stress

## Abstract

Diseases caused by pathogenic microorganisms in *Bombyx mori* have long been a major constraint on the sustainable development of sericulture. Current preventive strategies remain substantially constrained by issues of drug resistance and environmental compatibility. In recent years, the application of nanomaterials for pathogenic microorganism control has garnered escalating attention. Among these, chitosan–silver nanoparticles (CS-Ag NPs), as an emerging class of nanocomposites, integrate the biocompatibility and biodegradability of chitosan with the robust antimicrobial activity of silver nanoparticles, thereby exhibiting considerable potential for preventing pathogenic infections. Nevertheless, the efficacy of CS-Ag NPs against *B. mori* pathogens has not previously been documented. In this study, CS-Ag NPs were successfully synthesized via chemical reduction. Their antiviral activity was validated using quantitative PCR. The inhibitory efficacy of CS-Ag NPs against *Bacillus bombysepticus* and *Serratia marcescens* was evaluated through in vitro inhibition zone assays and bacterial growth curve analysis, with the minimum inhibitory (MIC) concentration for both pathogens determined. Notably, CS-Ag NPs exhibited no significant inhibitory effect on filamentous fungi, potentially due to the impaired ability of nanoparticles to penetrate fungal cell walls. Preliminary mechanistic investigations into the antimicrobial mechanism of CS-Ag NPs were conducted from the perspectives of oxidative stress. Our data showed that CS-Ag NPs could effectively alleviate ROS accumulation induced by the pathogen. In summary, our work systematically investigates the potential of CS-Ag NPs in controlling pathogens and enables the preliminary elucidation of their antibacterial mechanisms. These findings establish a theoretical foundation for the development of pharmaceuticals against pathogenic microorganisms and also offer novel insights into the ecofriendly management of diseases.

## Simple Summary

Diseases caused by viruses and bacteria are a major threat to silkworm (*Bombyx mori*) health and seriously limit silk production worldwide. Current disease control methods often rely on the extensive use of antimicrobial agents and chemical disinfectants, which contribute to antimicrobial resistance and environmental pollution, respectively. In this study, we explored a new, environmentally friendly approach using chitosan–silver nanoparticles, a material that combines a natural biopolymer with the antimicrobial properties of silver. We found that these nanoparticles effectively inhibited a key silkworm virus and two major bacterial pathogens, while showing no harmful effects on silkworm growth or cocoon production. Importantly, the nanoparticles improved silkworm survival during bacterial infection and helped reduce harmful oxidative stress in host cells. Although the nanoparticles were not effective against fungal pathogens, their selective activity reduces potential risks to non-target organisms. Overall, this study demonstrates that chitosan–silver nanoparticles are a promising and sustainable tool for controlling silkworm diseases, with potential benefits for environmentally responsible sericulture and agricultural disease management.

## 1. Introduction

The silkworm (*Bombyx mori*) is highly susceptible to a wide range of pathogenic microorganisms throughout its life cycle. These diseases not only severely compromise cocoon yield and quality but also constitute a major bottleneck restricting the sustainable development of the global sericulture industry. Among viral diseases, *B. mori* nucleopolyhedrovirus (BmNPV), a double-stranded circular DNA virus, is the primary etiological agent of silkworm nucleopolyhedrosis. Following oral infection, BmNPV replicates in the midgut epithelial cells of silkworm larvae and subsequently disseminates to multiple tissues, including the fat body, epidermis, and trachea, resulting in highly contagious outbreaks characterized by explosive mortality and significant reductions in cocoon production [1,2].

In addition to viral pathogens, silkworms are frequently threatened by fungal and bacterial infections. Entomopathogenic fungi such as *Beauveria bassiana* and *Metarhizium anisopliae* primarily invade the host through the integument, proliferate extensively within the hemocoel, and secrete secondary metabolites that disrupt immune function and induce systemic mycosis [3,4,5]. *Aspergillus flavus*, a facultative parasitic and saprophytic fungus ubiquitously distributed in silkworm rearing environments, produces highly potent mycotoxins that contaminate mulberry leaves and rearing facilities, leading to chronic intoxication manifested as growth retardation and developmental disorders in silkworms [6,7]. Among bacterial pathogens, *Bacillus bombysepticus*, a Gram-positive spore-forming bacterium, and *Serratia marcescens*, a Gram-negative opportunistic pathogen, are the principal causative agents of silkworm septicemia. Under conditions of elevated temperature and humidity, these bacterial diseases readily outbreak, with mortality rates commonly exceeding 50–80%, thereby imposing substantial economic losses on sericulture production [8,9,10,11,12,13,14,15].

The effective control of silkworm pathogens remains a core challenge for the sustainable development of the sericulture industry. Traditional chemical control strategies primarily rely on the application of agricultural antimicrobials and the periodic disinfection of rearing facilities. Although these approaches are cost-effective and operationally simple, increasing concerns regarding the emergence of antimicrobial resistance and the toxicity of persistent environmental residues have become increasingly prominent [16,17]. Biological control strategies, including microbial antagonists, phage-based therapies, and natural antimicrobial extracts, have been rapidly developed as environmentally friendly alternatives; however, their efficacy is often highly dependent on environmental conditions, and their stability and applicability require further improvement [14,18,19]. Disease-resistant silkworm breeding represents a fundamental strategy to enhance intrinsic host resistance, yet its widespread industrial application is constrained by long breeding cycles, high technical complexity, and ongoing public concerns regarding genetically modified organisms [7,19,20]. Consequently, the development of efficient, stable, and eco-friendly disease control strategies remains an urgent priority in sericulture.

In recent years, nanomaterials have emerged as innovative interdisciplinary tools with extensive application prospects in pest and disease management, owing to their nanoscale dimensions, high specific surface area, and favorable biocompatibility [21]. Previous studies have demonstrated that silver nanoparticles (AgNPs) not only exert significant inhibitory effects on BmNPV replication but also markedly suppress the growth and development of the microsporidian pathogen *Nosema bombycis* [22]. Nevertheless, the antimicrobial spectrum, targeting capability, biosafety, and underlying mechanisms of nanocomposite materials against major silkworm pathogens have not been systematically investigated and remain at a preliminary stage [23].

In order to develop an ideal antimicrobial agent for sericulture, the material must not only be effective against a wide range of pathogens, but also pose no threat to the silkworm host. Silver nanoparticles (Ag NPs) are widely recognized as one of the most potent metal-based antimicrobial and antiviral agents. Their unique, multi-target destructive mechanism effectively circumvents the development of pathogen resistance; however, their tendency to aggregate and their potential for off-target toxicity restrict their direct in vivo application. To address these issues, we selected chitosan (CS), a natural cationic polysaccharide derived from chitin, as the ideal complementary matrix. CS exhibits exceptional biodegradability, negligible toxicity to animal hosts and intrinsic antimicrobial properties. More importantly, its abundant protonated amino groups (-NH_3_^+^) confer a strong positive surface charge [24]. By synthesizing chitosan–silver nanocomposites (CS-Ag NPs), we have successfully integrated the unique properties of both materials into a highly synergistic architecture. In this system, the chitosan shell prevents silver aggregation and buffers its toxicity to protect silkworm development. Its cationic nature also acts as a ‘homing missile’ to facilitate targeted electrostatic binding to negatively charged pathogen membranes. This precise combination enables enhanced pathogen disruption and the controlled release of silver ions [25,26,27], making the nanocomposites an exceptionally rational and potent candidate for combating silkworm diseases. In recent years, CS-Ag NPs have demonstrated promising potential in agricultural disease management, postharvest preservation, and antiviral applications [28,29,30,31,32]. However, their application in silkworm disease control, particularly regarding in vivo protective efficacy and biosafety during silkworm development, remains largely unexplored.

Despite their immense potential, nanocomposites remain underutilized in sericulture due to a critical knowledge gap concerning their specific applications. This gap is characterized by a severe lack of systematic in vivo biosafety evaluations and multi-pathogen efficacy assessments within the delicate silkworm host. This study aimed to address this issue by systematically evaluating the antimicrobial potential of CS-Ag NPs against representative viral and bacterial pathogens of *B. mori*. Specifically, we investigated their antiviral activity against BmNPV and their antibacterial efficacy against *Bacillus bombysepticus* and *S. marcescens*. Crucially, we established a stringent in vivo toxicological safety baseline and demonstrated the robust protective effects of CS-Ag NPs at the level of the individual silkworm. Furthermore, we employed oxidative stress-related assessments to elucidate the underlying cytoprotective mechanisms. This work makes a significant contribution to the field by defining a clear operational window for nanomaterials in sericulture. It provides critical empirical evidence and a vital theoretical framework for developing next-generation, eco-friendly, precision nano-pharmaceuticals, offering a highly promising strategy for overcoming the limitations of traditional silkworm disease management.

## 2. Materials and Methods

### 2.1. Synthesis and Characterization of CS-Ag NPs

Chitosan–silver nanoparticles (CS-Ag NPs) were prepared via a two-step process involving in situ reduction followed by ionic gelation. First, 2 mg/mL of CS solution was prepared by dissolving chitosan (CS, molecular weight: 150 kDa, degree of deacetylation: ≥95%, purchased from Macklin Biochemical Co., Ltd., Shanghai, China) in 50 mL of 0.1% (*v*/*v*) acetic acid aqueous solution under continuous stirring. Then, 3.4 mg of silver nitrate (AgNO_3_) powder was directly added to the prepared CS solution to achieve a final AgNO_3_ concentration of 4 × 10^−4^ M, and the mixture was stirred continuously at 500 rpm and 95 °C for 30 min to obtain a homogeneous solution. Next, 45 μL of 1 mol/L NaOH was added to the reaction mixture to provide an alkaline environment, which accelerates the reduction of Ag^+^ to Ag^0^ by chitosan and simultaneously induces the macroscopic precipitation of the composite via chitosan deprotonation. This rapidly turned the solution yellow, indicating the successful formation of silver nanoparticles. After the solution cooled to room temperature, it was vacuum-filtered—a method selected for its high efficiency in rapidly separating these large, flocculant CS-Ag precipitates from the bulk solution—and the collected yellow precipitate was washed twice with deionized water to remove any residual NaOH or free silver ions. The filter cake was then dried in a constant-temperature oven at 60 °C for six hours to obtain the intermediate CS-Ag powder, which was stored at −80 °C for subsequent use [16,33]. To fabricate the final crosslinked CS-Ag nanoparticles (NPs), an aqueous solution of 0.1% (*v*/*v*) glacial acetic acid was prepared and adjusted to pH 4.8 using 50 µL of 1 mol/L NaOH. The previously synthesized CS-Ag powder was dissolved in this acetic acid solution to a concentration of 2 mg/mL. Next, 7.5 mL of this CS-Ag solution was placed under magnetic stirring and 2.5 mL of a sodium tripolyphosphate (TPP) solution (1.6 mg/mL) was added dropwise. The immediate onset of turbidity indicated the formation of the nanocomposites. After 30 min of continuous stirring, the mixture was purified by centrifugation (LC-LX-H185C, Shanghai Lichen Bangxi Instrument Technology Co., Ltd., Shanghai, China) at 16,000 rpm for 30 min at 4 °C. The supernatant was discarded and the pellet ultrasonically redispersed in deionized water. This washing and centrifugation cycle was repeated three times under identical conditions. Finally, the purified precipitate was uniformly dispersed in deionized water to yield the final CS-Ag NP suspension.

The physicochemical properties of the synthesized nanoparticles were systematically characterized using specific instrumental platforms. Optical absorption spectra were recorded using a Shimadzu UV-2550 UV-Vis spectrophotometer (Kyoto, Japan) in the 300–600 nm wavelength range. For UV-Vis measurements, the purified nanoparticle suspensions were diluted with deionized water to the appropriate concentration and analyzed in standard quartz cuvettes. The internal morphological features and core–shell structures were observed via transmission electron microscopy (TEM; HT-7800, Hitachi, Tokyo, Japan). TEM samples were prepared by ultrasonically dispersing the nanoparticle suspension for 10 min using an ultrasonic cleaner (SK2210HP, Shanghai Kudos, Shanghai, China), subsequently drop-casting 10 µL of the highly diluted suspension onto carbon-coated copper grids and allowing them to air-dry naturally at room temperature. The 3D surface morphology and aggregation state were also evaluated using a high-resolution field-emission scanning electron microscope (FE-SEM; Regulus 8100, Hitachi, Tokyo, Japan), operating at 5.0 kV. The SEM samples were prepared by dropping the diluted suspension onto a clean silicon wafer, allowing it to dry completely at room temperature and then sputter-coating the surface with a thin layer of gold to enhance electrical conductivity. Finally, the hydrodynamic diameter distributions and surface charge (zeta potential) of the nanoparticles were measured using a Zetasizer Nano ZS instrument (Malvern Panalytical, Malvern, UK) at a constant temperature of 25 °C. Prior to DLS and zeta potential measurements, the samples were diluted extensively in deionized water and sonicated gently for five minutes to ensure uniform dispersion and avoid multiple light scattering effects.

### 2.2. Cell Proliferation and Viability Assay (CCK-8 Assay)

The *B. mori* ovary-derived cell line (BmN) was subcultured and maintained by the Silkworm Pathology Laboratory, Sericultural Research Institute, Chinese Academy of Agricultural Sciences (CAAS). This cell line was routinely cultured in TC-100 insect medium, which was supplemented with 10% (*v*/*v*) fetal bovine serum (FBS) and 1% penicillin-streptomycin. The cells were maintained in an incubator at 27 °C. For the viability assay, BmN cells in the logarithmic growth phase were harvested and seeded into 96-well plates at a density of 1 × 10^3^ cells per well. After achieving approximately 70–80% confluency, the cells were treated with serial concentration gradients of CS-Ag NPs and incubated at 27 °C for 24 h. Subsequently, 10 µL of CCK-8 reagent was added to each well. The 96-well plate was then incubated at 27 °C for 4 h. The optical density (OD) value was measured at 450 nm using a microplate reader (SpectraMax i3, Molecular Devices, San Jose, CA, USA).

### 2.3. Absolute Quantitative PCR Assay

In order to evaluate the antiviral efficacy of CS-Ag NPs, a BmNPV replication inhibition assay was performed using the *B. mori* ovary-derived BmN cells. The cells were seeded into 6-well plates and cultured to approximately 80% confluency. The cells were then inoculated with BmNPV at a specific multiplicity of infection (MOI). Subsequent to a 2-h viral adsorption period, the viral inoculum was removed. For the treatment group, the cells were subsequently cultured in fresh medium containing 100 µg/mL CS-Ag NPs. For the control group, the cells were cultured in fresh medium devoid of nanoparticles. At 24, 36, and 48 h post-infection (hpi), BmN cells from each experimental group were harvested, and total genomic DNA was extracted using the SanPrep Column DNA Extraction Kit (Sangon Biotech (Shanghai) Co., Ltd., Shanghai, China). DNA concentration and purity were quantified with a NanoDrop 2000 spectrophotometer (Thermo Fisher Scientific, Waltham, MA, USA) and samples were normalized to a standard concentration for subsequent analyses. The BmNPV core gene *ie-1* (GenBank: NP_047491.1) was selected as the target gene. A standard curve was constructed by preparing serial dilutions (10^1^–10^8^ copies/µL) of a plasmid standard containing the *ie-1* fragment. Quantitative PCR reactions were performed using the ChamQ SYBR Master Mix system (Vazyme Biotech Co., Ltd., Nanjing, China). The thermal cycling protocol was as follows: initial denaturation at 94 °C for 30 s; followed by 40 cycles of denaturation at 94 °C for 5 s and annealing/extension at 60 °C for 30 s. Post-amplification melting curve analysis was conducted to verify amplicon specificity. Each experimental group was analyzed with three biological replicates and three technical replicates. Viral genome copy numbers were calculated using the standard curve method. The primers used in this study were presented in Supplementary Materials.

### 2.4. Determination of Antimicrobial Activity via Disk Diffusion Assay

All pathogens used in this study, including BmNPV, *B. bombysepticus*, *S. marcescens*, *Beauveria bassiana*, *Metarhizium anisopliae*, and *Aspergillus flavus*, were isolated, purified, and maintained by the Silkworm Pathology Laboratory, Sericultural Research Institute, CAAS. Bacterial strains (*S. marcescens*, *B. bombysepticus*) were inoculated into LB liquid medium and cultured at 30 °C with shaking at 180 rpm until reaching the logarithmic growth phase. The bacterial suspension concentration was adjusted to approximately 1 × 10^8^ CFU/mL, and 100 µL aliquots were uniformly spread onto LB agar plates. Mature spores of *Beauveria bassiana*, *Metarhizium anisopliae*, and *Aspergillus flavus* were prepared as spore suspensions using a sterile 0.05% Tween-80 solution. After adjusting the concentration to 1 × 10^6^ spores/mL, 100 µL of the suspension was uniformly inoculated onto the surface of PDA medium. Sterile filter paper disks were impregnated with CS-Ag NPs at different concentrations. Equivalent concentrations of CS NPs and sterile water served as control groups. Bacterial plates were incubated at 30 °C for 18 h; fungal plates were incubated at 28 °C for 2–5 days until mycelial coverage was observed on control plates. The inhibition zone diameter (IZD) was measured for each group. Each experiment was independently replicated three times (n = 3).

### 2.5. Pathogen Growth Kinetics and Minimum Inhibitory Concentration (MIC) Assay

Bacterial suspensions of *S. marcescens* and *B. bombysepticus* were adjusted to 1 × 10^6^ CFU/mL, respectively, and thoroughly mixed with CS-Ag NPs at different final concentrations. Equivalent concentrations of pure CS NPs and sterile water served as control groups. All groups were incubated at 30 °C. The OD_600_ value of each group was measured every 1–2 h using a microplate reader or spectrophotometer. Growth kinetic curves were plotted with time on the x-axis and OD_600_ value on the y-axis. The MIC was defined as the lowest concentration of the test material at which no significant bacterial growth was visually observed (or the OD value growth rate was <5%) after 24 h of incubation.

### 2.6. Safety Assessment of CS-Ag NPs on Silkworm Growth

Test silkworms (strain P50, provided by the Silkworm Resource Conservation Center, Sericultural Research Institute, CAAS) were reared under controlled conditions of 28 °C, 60–80% relative humidity, and a 16 h:8 h light-dark cycle, with ad libitum feeding of fresh mulberry leaves until the initiation of the 5th instar stage. Healthy 5th-instar P50 larvae were randomly allocated into 4 experimental groups, with 45 larvae per group and three biological replicates (15 larvae per replicate), Baseline average body weight exhibited no statistically significant differences among groups. CS-Ag NPs and sterile water were uniformly applied to the surface of fresh mulberry leaves. The leaves were sectioned into standardized 4 cm × 4 cm segments, and each group received an equivalent quantity of treated leaves per feeding until cocoon formation. The blank control group was administered leaves treated with an equal volume of deionized water. Three experimental groups were established with CS-Ag NPs additive concentrations of 100 µg/g, 200 µg/g, and 300 µg/g per larva, respectively. Daily larval body weights were recorded, and comparative analyses of daily weight gain and cocoon weight were performed between treatment and control groups using appropriate statistical methods.

### 2.7. Evaluation of the In Vivo Protective Efficacy of CS-Ag NPs Against S. marcescens Infection in Silkworms

The experiment was designed with two experimental groups (45 silkworms per group) and 3 independent biological replicates. Group A: Silkworms were administered mulberry leaves coated with a specific safe dosage of 300 µg/g of CS-Ag NPs for 3 consecutive days to establish an in vivo defense barrier. Group B: Silkworms were fed an equivalent quantity of mulberry leaves treated with deionized water. Silkworms in both groups were inoculated via abdominal intersegmental injection with 10 µL of *S. marcescens* at the LD_50_ concentration. Survival status of silkworms in each group was continuously monitored for 96 h post-inoculation. Survival curves were constructed using the Kaplan–Meier method [34].

### 2.8. Determination of Intracellular Reactive Oxygen Species (ROS) Levels

To elucidate the cytoprotective mechanism of the synthesized nanocomposites, an intracellular reactive oxygen species (ROS) assay was performed to evaluate whether CS-Ag NPs could mitigate oxidative stress induced by pathogens. BmN cells were seeded into 6-well plates at a density of 1 × 10^6^ cells per well and cultured until they reached the required level of confluency. The experiment comprised a pathogen-infected control group and a nanocomposite treatment group. For the treatment group specifically, the cells were pre-treated with 100 µg/mL of CS-Ag NPs and then inoculated with *S. marcescens* to induce intracellular oxidative stress. Subsequent to the designated incubation period, the culture medium was removed. The level of intracellular reactive oxygen species (ROS) was determined by means of the DCFH-DA fluorescent probe. Each well was then supplemented with DCFH-DA at a final concentration of 10 μmol/L and incubated in a 27 °C dark incubator for 30 min [35]. Subsequent to the process of incubation, the cells were subjected to a washing procedure comprising three cycles of incubation in serum-free medium, with the objective of the removal of any extracellular probes. Fluorescence intensity was then measured using a multifunctional microplate reader with an excitation wavelength set at 488 nm and an emission wavelength set at 525 nm.

### 2.9. Statistical Analysis

All quantitative experimental data are expressed as mean ± standard error (Mean ± SE). Statistical analyses were performed using *t*-tests in GraphPad Prism version 8.0.2 (GraphPad Software, San Diego, CA, USA). Statistical significance was set at a *p* value < 0.05. Significance levels are denoted as follows: * for *p* < 0.05; ** for *p* < 0.01; *** for *p* < 0.001; **** for *p* < 0.0001; ns indicates no significant difference.

## 3. Results

### 3.1. Physicochemical Characterization of CS NP and CS-Ag NPs

**Figure 1:**
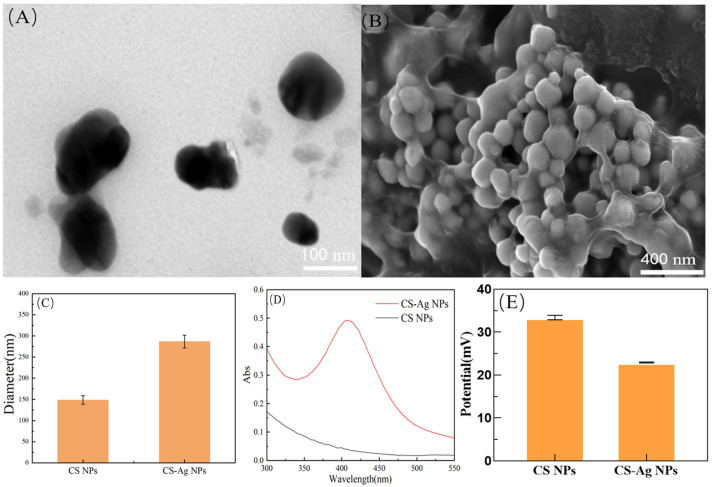
Physicochemical characterization of CS NPs and CS-Ag NPs. (**A**) Transmission electron microscopy (TEM) image of CS NPs. (**B**) Scanning electron microscopy (SEM) image of CS-Ag NPs. (**C**) Average hydrodynamic diameters of CS NPs and CS-Ag NPs measured by dynamic light scattering (DLS). (**D**) UV-Vis absorption spectra of CS NPs and CS-Ag NPs. (**E**) Average zeta potentials of CS NPs and CS-Ag NPs.

We observed the morphology and size of the nanoparticles using a transmission electron microscopy (TEM). The CS NPs displayed a homogeneous spherical morphology with excellent dispersibility and minimal aggregation, exhibiting an average particle size of approximately 98 nm (Figure 1A). Furthermore, as explicitly revealed by scanning electron microscopy (SEM) analysis, the CS-Ag NPs exhibited a well-defined core–shell architecture, wherein a high-electron-density metallic silver core (Ag NPs) was tightly encapsulated within a low-electron-density chitosan matrix (CS shell). This in situ encapsulation configuration not only confers effective steric stabilization to mitigate Ostwald ripening of the silver nanoparticles but also results in a concomitant increase in the apparent particle size to approximately 220 nm (Figure 1B). The hydrodynamic diameter of the nanoparticles was determined via dynamic light scattering (DLS), further verifying the reliability of particle size measurements. The hydrodynamic diameter of CS NPs was 148 nm, whereas that of CS-Ag NPs reached 282 nm (Figure 1C). The values measured by DLS were generally larger than the physical particle sizes determined by TEM, which is attributed to the swelling effect of chitosan molecular chains in the aqueous phase and the presence of a surface hydration layer. The substantial size augmentation observed in CS-Ag NPs further corroborates the successful immobilization and structural expansion of silver nanoparticles within the chitosan matrix. As shown in Figure 1D, CS-Ag NPs exhibited a prominent and well defined absorption peak within the 400–410 nm range, a spectral signature characteristic of the Localized Surface Plasmon Resonance (LSPR) of silver nanoparticles [36]. The emergence of this peak precludes the occurrence of physical doping and confirms the stable crystalline state of silver. In contrast, CS NPs remained optically transparent across this wavelength interval, lacking any discernible characteristic absorption. Integration of morphological analysis, particle size distribution, and spectroscopic characterization confirmed the successful fabrication of physically stable, morphologically uniform CS-Ag NPs.

To evaluate the stability and surface characteristics of CS NPs and CS-Ag NPs in aqueous dispersion, zeta potential measurements were performed. The results demonstrated that CS NPs exhibited a substantially positive surface charge, with a measured zeta potential of +33.45 mV. This result confirms that the abundant amino groups (-NH_2_) on the chitosan backbone undergo protonation under acidic conditions, forming -NH_3_^+^ that confer significant positive charge characteristics to the nanoparticles. The relatively high positive potential also suggests that CS NPs possess favorable electrostatic stability and dispersibility. In contrast, the Zeta potential of CS-Ag NPs exhibited a slight reduction to +23.58 mV. This charge attenuation is primarily ascribed to the electrostatic interaction between Ag NPs and the -NH_3_^+^ of chitosan during CS-Ag NPs synthesis. Despite this decrease, the zeta potential of +23.58 mV still remains above the critical stability threshold (>±20 mV) commonly recognized in colloidal systems (Figure 1E).

### 3.2. Inhibitory Effects of CS-Ag NPs on the Replication of B. mori Nucleopolyhedrovirus

**Figure 2:**
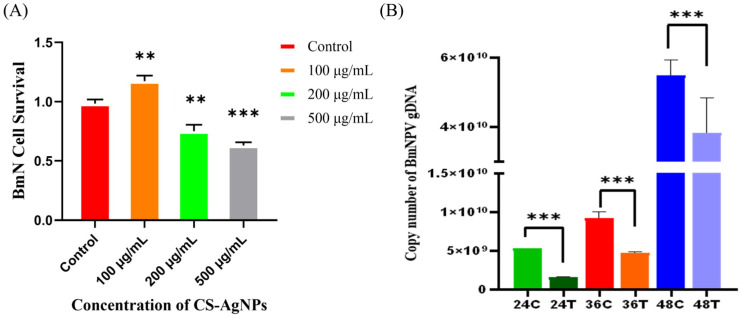
Cytotoxicity and antiviral activity of CS-Ag NPs in BmN cells. (**A**) Effect of CS-Ag NPs on proliferation viability of BmN cells, (**B**) qPCR results showing the inhibitory effects of CS-Ag NPs on BmNPV replication. On the X-axis, the numbers 24, 36, and 48 indicate the hours post-infection (hpi); “C” represents the untreated control group, and “T” represents the CS-Ag NP treatment group. Data are presented as mean ± SD from three independent biological replicates (** for *p* < 0.01; *** for *p* < 0.001).

We first assessed the safety of CS-Ag NPs in *B. mori* BmN cells using the CCK-8 cytotoxicity assay. The results showed that CS-Ag NPs exerted a distinct biphasic dose–response effect on BmN cells. At a concentration of 100 µg/mL, the viability of BmN cells showed a significant increasing trend compared to the control group. This might be due to the chitosan component and low concentrations of silver nanoparticles synergistically activating cellular stress defense pathways and enhancing metabolic activity. With further elevation of CS-Ag NPs concentration to 200 µg/mL and 500 µg/mL, cell viability decreased significantly. Based on this result, the maximum working concentration of CS-Ag NPs was standardized to ≤100 µg/mL for all subsequent in vitro and in vivo experimental assays (Figure 2A).

The effect of CS-Ag NPs on BmNPV was evaluated in BmN cells. As shown in Figure 2B, quantitative real-time PCR analysis indicated that in the untreated control group, the copy number of the critical gene *ie-1* rapidly increased tenfold, rising from 5.31 × 10^9^ copies/µL at 24 h post-infection (hpi) to 5.52 × 10^10^ copies/µL by 48 hpi. Conversely, the CS-Ag NP treatment group showed a statistically significant suppression of viral load at all monitored time points (Figure 2B).

### 3.3. Antibacterial Activity of CS-Ag NPs Against B. bombysepticus

**Figure 3:**
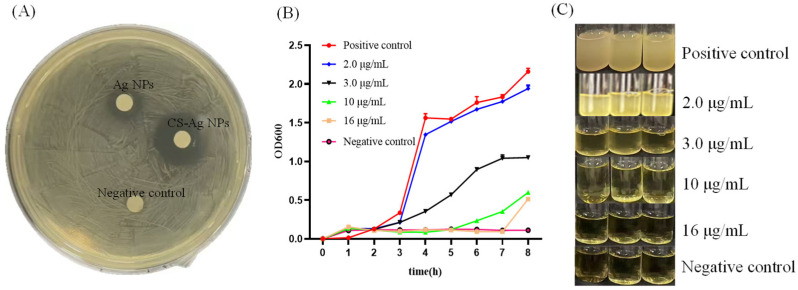
In vitro antibacterial activity and MIC determination of CS-Ag NPs against *B. bombysepticus*. (**A**) Antibacterial effects of different materials. (**B**) Bacterial growth curve (**C**) Determination of minimum inhibitory concentration. Data are presented as mean ± SD from three independent biological replicates.

The antimicrobial efficacy of CS NPs, Ag NPs, and CS-Ag NPs against *B. bombysepticus* was systematically assessed. Comparative analysis demonstrated that 10 µg/mL CS NPs alone exhibited marginal bacteriostatic activity against *B. bombysepticus*, whereas Ag NPs at equivalent concentrations displayed potent antibacterial efficacy. Notably, CS-Ag NPs exhibited the most potent inhibitory activity, as evidenced by the largest inhibition zone diameter (Figure 3A). Subsequently, a series of concentration gradients of CS-Ag NPs were established to determine the MIC against *B. bombysepticus*. Experimental results revealed that at a concentration of 2 µg/mL, the turbidity of the bacterial culture was comparable to that of the control group, indicating the absence of antibacterial activity at this concentration. At 3 µg/mL, a reduction in turbidity was observed. Upon further increasing the concentration to 10 µg/mL, the turbidity decreased significantly, preliminarily suggesting that this concentration was proximate to the antibacterial threshold. At 16 µg/mL, the bacterial culture remained clear throughout the observation period, with no detectable signs of bacterial proliferation (Figure 3B). The MIC value was further confirmed by bacterial growth curve experiments. As shown in Figure 3C, 2 µg/mL CS-Ag NPs showed no inhibitory effect on *B. bombysepticus* growth. A concentration of 3 µg/mL CS-Ag NPs partially inhibited growth, but the bacteria still entered the logarithmic growth phase after 4 h of culture at this concentration, with the OD600 value gradually increasing. When the CS-Ag NPs concentration was increased to 10 µg/mL, the OD600 value remained stable at the baseline level. At a concentration of 16 µg/mL, the OD600 value was consistent with the control group (Figure 3C). Consequently, the MIC of CS-Ag NPs against *B. bombysepticus* was determined to be 10 µg/mL.

### 3.4. Antibacterial Activity of CS-Ag NPs Against S. marcescens

**Figure 4:**
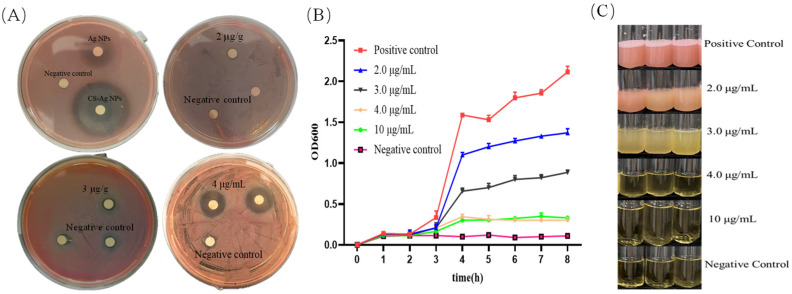
In vitro antibacterial activity and MIC determination of CS-Ag NPs against *S. marcescens*. (**A**) Antibacterial effects of different materials and Preliminary screening of MIC for CS-Ag NPs against *S. marcescens*. (Note: To avoid visual clutter, not all inhibition zones are labeled on the photographs; the unlabeled filter paper disks within each Petri dish represent technical replicates of the corresponding treatment.) (**B**) Bacterial growth curve. (**C**) Determination of minimum inhibitory concentration. Data are presented as mean ± SD from three independent biological replicates.

As a major opportunistic pathogen inducing septicemia in silkworms, the rapid proliferation of *S. marcescens* frequently results in the collapse of the host immune defense system. We conducted a comparative analysis on the effects of CS NPs, Ag NPs, and CS-Ag NPs against *S. marcescens*. CS NPs and Ag NPs at 10 µg/mL exhibited only limited inhibition zones, whereas CS-Ag NPs displayed significantly enhanced antibacterial activity (Figure 4A). Subsequently, a series of concentration gradients of CS-Ag NPs were established to determine the MIC against *S. marcescens*. At concentrations of 2 µg/mL and 3 µg/mL, both the turbidity of bacterial cultures and the diameter of inhibition zones were comparable to those of the control group, indicating an absence of antibacterial activity. At 4 µg/mL, the turbidity decreased significantly while the inhibition zone diameter increased markedly, preliminarily suggesting that this concentration approximated the antibacterial threshold. When the CS-Ag NPs concentration was further elevated to 10 µg/mL, the bacterial culture remained clear with no observable proliferation (Figure 4A,B). The MIC was further confirmed through bacterial growth curve assays. Within the concentration range of 2.0–3.0 µg/mL, although *S. marcescens* exhibited an extended lag phase, it subsequently entered the logarithmic growth phase and achieved a high plateau OD value. When the CS-Ag NPs concentration was elevated to 4.0 µg/mL and 10 µg/mL, bacterial growth was completely inhibited, with the OD600 value remaining at baseline throughout the 8-h real-time monitoring period (Figure 4C). Consequently, we determined that the MIC of CS-Ag NPs against *S. marcescens* is 4.0 µg/mL.

### 3.5. CS-Ag NPs Have No Obvious Inhibitory Activity Against the Pathogenic Fungi of Silkworms

**Figure 5:**
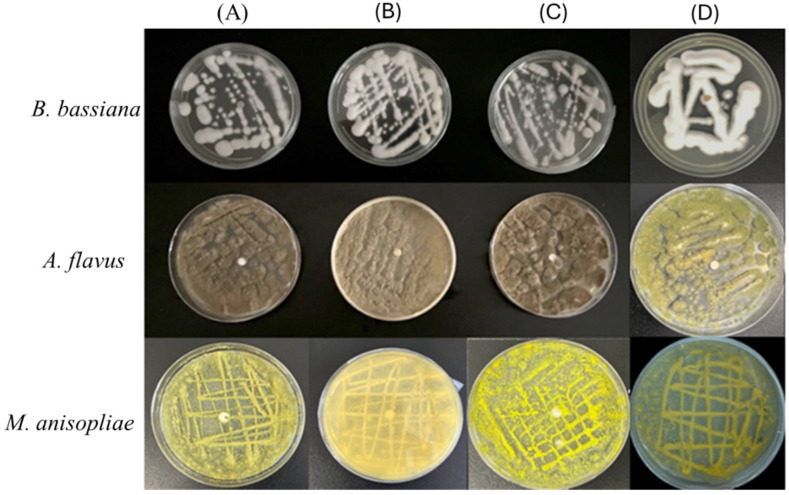
In vitro antifungal activity of CS-Ag NPs against *B. mori* pathogenic fungi. Panels from top to bottom represent *Beauveria bassiana*, *Aspergillus flavus*, and *Metarhizium anisopliae*, respectively. (**A**–**C**) Three independent biological replicates of the experimental group treated with 100 µg/mL CS-Ag NPs. (**D**) Representative image of the negative control group. Data are presented as mean ± SD from three independent biological replicates.

To further characterize the antimicrobial spectrum of CS-Ag NPs, we evaluated the inhibitory effects of CS-Ag NPs against three common silkworm pathogenic fungi (*Beauveria bassiana*, *Metarhizium anisopliae*, and *Aspergillus flavus*). Experimental results demonstrated that even at a CS-Ag NPs concentration of 100 µg/mL, no significant differences in colony morphology or radial growth rate were observed between the experimental and control groups. These results indicate that CS-Ag NPs lack the anticipated inhibitory activity against these filamentous fungi, thereby exhibiting pronounced antimicrobial selectivity (Figure 5).

### 3.6. No Adverse Effect of CS-Ag NPs on Silkworm Growth

**Figure 6:**
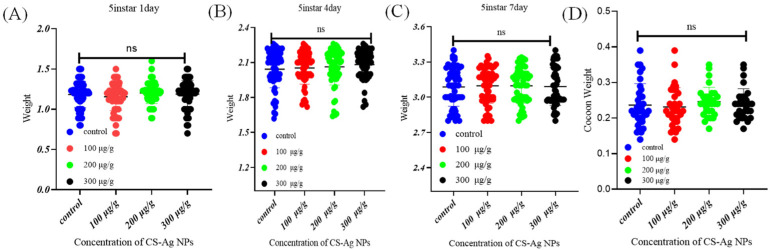
Effects of CS-Ag NPs on the daily growth (**A**–**C**) and cocoon weight (**D**) of *B. mori*. Data are presented as mean ± SD from three independent biological replicates (ns indicates no significant difference).

To systematically assess the biosafety profile of the nanomaterial, we evaluated the impacts of graded dietary supplementation concentrations of CS-Ag NPs on the growth performance, developmental dynamics, and economic characteristics of fifth-instar silkworms. The experimental results showed that, in comparison with the blank control group, dietary supplementation of different concentrations of CS-Ag NPs exerted no statistically significant effect on the daily weight gain of silkworms. These results indicates that CS-Ag NPs do not interfere with the digestion and absorption of mulberry leaf nutrients in the silkworm (Figure 6A–C). As a critical indicator for evaluating pesticide or nanomaterial toxicity, we further examined cocooning performance. Experimental data showed that even at a dosage of 300 µg/g, silkworms were able to normally complete spinning and cocooning processes and enter the metamorphosis stage, with no observed prolongation of the life stage or developmental delay. The key economic indicators such as whole cocoon weight, cocoon shell weight, and cocooning rate remained consistent across all groups (Figure 6D).

The establishment of this toxicological safety baseline and the identification of the maximum tolerated dose (up to 300 µg/g) were critical prerequisites for the subsequent in vivo therapeutic evaluations.

### 3.7. Assessment of Survival Efficacy of Prophylactic CS-Ag NPs Intervention in Preventing S. marcescens Infection in Silkworms

**Figure 7:**
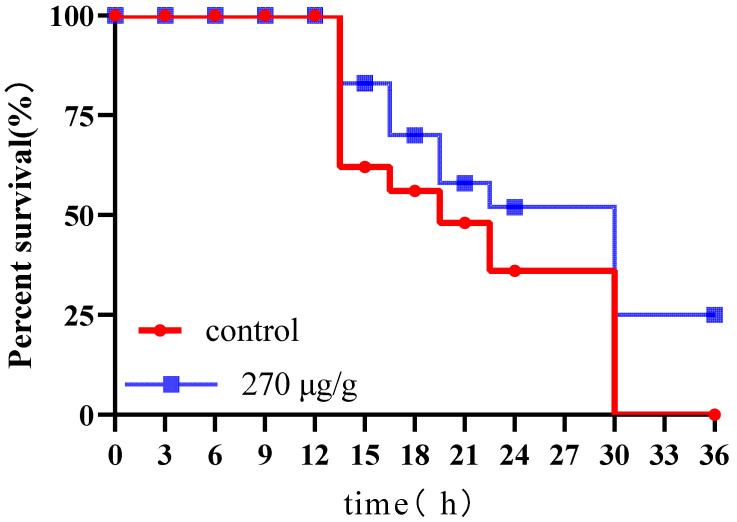
Survival curves of *B. mori* infected with *S. marcescens* after dietary supplementation with chitosan–silver nanoparticles. Data are presented as mean ± SD from three independent biological replicates.

To evaluate the protective efficacy of oral CS-Ag NPs against *S. marcescens* infection in silkworms, survival curves were constructed using the Kaplan–Meier statistical method [34]. Experimental data showed that following *S. marcescens* inoculation, the infection control group (Group B) exhibited a mortality rate approaching 100%, with the mortality peak concentrated between 12–30 hpi. The survival rate declined to 0% by 36 hpi, indicating that the pathogen induced a systemic defensive collapse in the host organism. In contrast, the CS-Ag NPs prophylactic group significantly altered this pathological trajectory. At the early infection stage (12 hpi), the survival rate increased from 60.0% in the control group to 78.2%, preliminarily suppressing the initial pathogenic outbreak. During the mid-stage (24 hpi), the treatment group maintained a 53.4% survival rate, effectively delaying septicemia progression. The most pronounced divergence was observed at 36 hpi, while the control group exhibited complete mortality, the CS-Ag NP treatment group retained 25.0% viable individuals. These results indicate that CS-Ag NPs not only prolonged survival time but also enabled a subset of silkworms to surpass the lethal infection threshold (Figure 7).

### 3.8. CS-Ag NPs Mitigate Oxidative Stress Induced by S. marcescens Infection

**Figure 8:**
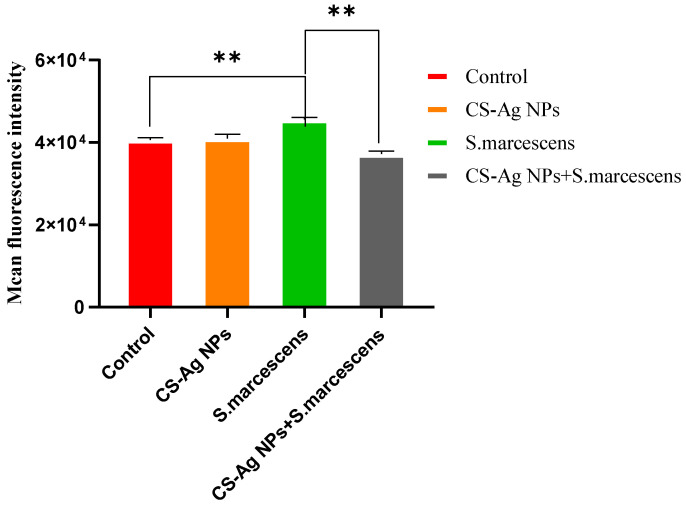
Measurement of intracellular ROS levels in BmN cells under different treatments. The cells were either left untreated (Control), treated with 100 µg/mL CS-Ag NPs alone, infected with *S. marcescens* alone, or pre-treated with 100 µg/mL CS-Ag NPs followed by *S. marcescens* infection. Data are presented as mean ± SD from three independent biological replicates (** for *p* < 0.01).

Pathogen infection often induces the overproduction of ROS in host cells, leading to oxidative stress damage. To evaluate the cytoprotective potential of CS-Ag NPs, intracellular ROS levels were quantified in BmN cells across distinct treatment groups. Experimental data showed that 100 µg/mL CS-Ag NPs exerted no significant impact on intracellular ROS levels. In contrast, inoculation with *S. marcescens* elicited a significant ROS burst in BmN cells. Notably, co-treatment with 100 µg/mL CS-Ag NPs and *S. marcescens* led to the restoration of intracellular ROS levels to a range comparable to that of the untreated control group (Figure 8).

## 4. Discussion

The sustainable development of sericulture is constantly threatened by severe microbial diseases. Conventional chemical agents are widely used, but increasing concerns over environmental toxicity and pathogen resistance highlight the urgent need for safer, innovative anti-infective strategies. In this study, we successfully developed a new class of antimicrobial agent, namely, chitosan–silver nanocomposites (CS-Ag NPs), and demonstrated their potent efficacy against major pathogens of *B. mori*, in addition to excellent in vivo biocompatibility. The superior performance of these nanocomposites is fundamentally rooted in their synergistic physicochemical properties.

The physicochemical characterizations confirmed the successful construction of the CS-Ag NPs composite system. This system was specifically designed to overcome the inherent limitations of its individual components. While free silver nanoparticles (Ag NPs) have been shown to possess strong bactericidal activity, they have also been observed to exhibit a propensity for aggregation in complex biological microenvironments, such as the neutral-pH midgut of silkworms. The nonselective toxicity of free silver ions remains an unignorable concern. In contrast, pure chitosan (CS), despite its inherent antimicrobial properties mediated by protonated amino groups (-NH_3_^+^) [37], frequently exhibits inadequate charge density or effective membrane-binding sites to profoundly disrupt bacterial lipid bilayers when used independently [38]. In synthesized CS-Ag NPs, transmission electron microscopy (TEM) and UV-Vis spectroscopy (which revealed an LSPR peak at 400–410 nm) [36] confirmed a distinct core–shell architecture [39]. This structure employs the low-electron-density CS matrix as a steric stabilizer, thereby effectively encapsulating the high-electron-density Ag core. This process serves to prevent aggregation, while concomitantly mitigating the biotoxicity of silver through physical confinement [40].

In addition, the CS-Ag NPs demonstrated a highly positive surface charge, as indicated by Zeta potential analysis, which suggests inherent electrostatic targeting capabilities. The abundant amino and hydroxyl functional groups of the CS shell facilitate electrostatic neutralization and tight binding to the negatively charged bacterial membrane. This interaction induces localized membrane depolarization and pore expansion, thereby lowering the energy barrier for the sustained release of Ag+ ions to penetrate the cell envelope. Consequently, this synergistic mechanism not only ensures excellent colloidal stability but also drives the potent disruption of intracellular metabolic cascades, establishing a robust structural foundation for their successful application in the complex physiological microenvironment of *B. mori*.

In this study, we demonstrated that CS-Ag NPs significantly downregulate the transcriptional expression of *ie-1*, indicating potent antiviral activity. The antiviral mechanism is hypothesized to primarily involve the synergistic effects of physical barrier formation and chemical interference. Due to the high density of positive surface charges on CS-Ag NPs, they effectively adsorb negatively charged viral envelope proteins through electrostatic interactions, thus physically preventing viral attachment and membrane fusion with BmN cells [41]. The release of Ag^+^ by CS-Ag NPs with high nucleophilicity allows it to penetrate the viral envelope and subsequently bind to viral genomic DNA/RNA. This interaction disrupts nucleic acid base pairing and interferes with the binding of transcription factors to promoter regions, thereby specifically inhibiting viral cascade transcription [42].

It is imperative to note that the biological models utilized for antiviral and antibacterial evaluations in this study are distinct (in vitro BmN cells for BmNPV and in vivo *B. mori* larvae for bacterial pathogens). It is important to note that the experimental design was not intended for a direct, head-to-head comparison of CS-Ag NPs’ efficacy across different pathogen kingdoms. Instead, the BmN cell line was strategically employed to precisely quantify the absolute copy number of the viral *ie-1* gene at the molecular level, thus isolating the direct interference of the nanomaterial from the complex systemic immune responses of the host. Conversely, pathogens such as *S. marcescens* characteristically invade via the gut to induce systemic septicemia; consequently, evaluating the protective efficacy necessitates the context of an intact intestinal barrier and systemic immunity, rendering the in vivo larval model indispensable. This multi-tiered screening approach is a comprehensive method of analyzing the versatile application potential of CS-Ag NPs from both molecular and organismal perspectives.

Recurrent outbreaks of microbial diseases caused by bacteria, fungi, and viruses, lead to substantial reductions in cocoon yield and quality, typically resulting in economic losses ranging from 30% to 60% [43]. Traditional chemical control strategies are confronted with critical limitations, including the development of drug resistance and environmental persistence [44]. In contrast, nanomaterials, characterized by their high specific surface area, unique physicochemical properties, and broad-spectrum antimicrobial activity, offer a promising alternative for addressing this challenge [45]. Although CS-Ag NPs have exhibited potent antimicrobial efficacy in various applications, research on the utilization of CS-Ag NPs for the control of major silkworm pathogens remains in the preliminary stages. Our research systematically evaluated the intervention efficacy of CS-Ag NPs against two core pathogenic bacteria in silkworms. In vitro antibacterial assays confirmed that the diameter of the inhibition zone of CS-Ag NPs was significantly larger than that of individual components at equivalent doses, demonstrating a marked synergistic advantage of CS-Ag NPs in inhibiting pathogens. High resolution dynamic growth curve analysis revealed that CS-Ag NPs exhibited distinct dose dependent inhibitory effects, with heterogeneous sensitivity profiles observed across different pathogens. For *S. marcescens* (Gram-negative), CS-Ag NPs showed exceptional inhibitory sensitivity. The MIC was precisely determined at 4.0 µg/mL, and bacterial proliferation was completely abrogated at this concentration. For *B. bombysepticus* (Gram-positive), exposure to 3.0 μg/mL of CS-Ag NPs resulted in a significant prolongation of the lag phase, while the MIC required to achieve complete growth inhibition was determined to be 10.0 µg/mL. This differential sensitivity is attributed to the physical barrier function of the thick peptidoglycan layer in Gram-positive bacteria, which confers resistance to nanomaterial penetration. The superior antibacterial efficacy of CS-Ag NPs arises from the interplay between their distinctive physicochemical properties and the bacterial biointerface. The high positive surface potential of CS-Ag NPs generates strong electrostatic interactions with the negatively charged cell membranes of pathogens, facilitating efficient biointerface anchoring that directly compromises membrane integrity. Additionally, the chitosan scaffold not only provides structural support for the nanosilver component but also modulates Ag^+^ release kinetics, enabling the maintenance of therapeutically effective local bactericidal concentrations over an extended duration [46,47]. Furthermore, the composite architecture significantly mitigates the self-aggregation propensity of Ag NPs in aqueous matrices, thereby maximizing their effective surface area. This enhancement in surface availability increases pathogen receptor interaction frequency and substantially reduces the minimal inhibitory dosage requirements [48].

Subsequent to confirming the potent bactericidal and antiviral properties of CS-Ag NPs, this study further evaluated their inhibitory efficacy against common filamentous fungal pathogens affecting silkworms, including *Beauveria bassiana*, *Metarhizium anisopliae*, and *Aspergillus flavus*. Our results demonstrated that even at an elevated CS-Ag NP treatment concentration of 100 µg/mL, no statistically significant differences were observed in colony growth kinetics or final diameter between the experimental and control groups. This result deviates from the previously reported antifungal efficacy of Chitosan@CuO and ZnO-CS [49], thereby highlighting the pathogen-specific intervention characteristics of distinct hybrid nanosystems [50]. The underlying mechanism for this phenomenon may be attributed to the structural complexity of the fungal cell wall, which differs fundamentally from the simple peptidoglycan layer or lipopolysaccharide (LPS) outer membrane of bacteria. Specifically, the fungal cell wall comprises a dense, cross-linked network structure composed of β-1,3-glucan, chitin, and mannoproteins. Despite the ability of CS-Ag NPs to adhere to bacterial surfaces via electrostatic interactions, their dimensions may be insufficient to penetrate the dense composite wall layer of fungi and reach the cell membrane. Furthermore, as eukaryotic organisms, filamentous fungi possess sophisticated antioxidant enzyme systems and highly developed intracellular transport networks [51]. Even if trace amounts of Ag^+^ penetrate the cell wall, they are likely to be rapidly chelated or expelled via efflux pumps, thereby maintaining intracellular redox homeostasis [52].

The multifaceted protective effects of CS-Ag NPs—which range from the suppression of bacterial proliferation to the mitigation of pathogen-induced oxidative stress—can be unified by their physicochemical bio-interface interactions. The dense positive charges of the chitosan shell facilitate rapid electrostatic adherence to negatively charged bacterial membranes, causing structural disruption. In addition, the composite acts as a dynamic shield for host cells. As demonstrated by our intracellular ROS assessment, the pre-treatment with CS-Ag NPs effectively neutralized the pathogen-induced ROS burst in BmN cells. This finding indicates that, in addition to direct bactericidal and antiviral activities, CS-Ag NPs may also impede the action of virulence factors at the host–pathogen interface, thereby contributing to the maintenance of host redox homeostasis and the prevention of systemic immune collapse.

From a practical sericulture perspective, the synergistic roles of Ag and chitosan mean that CS-Ag NPs can be used as an effective prophylactic feed additive by spraying mulberry leaves, or as an environmentally friendly disinfectant for rearing facilities. However, to provide a balanced view of their potential uses, the limitations and conditional factors affecting their efficacy must be acknowledged. Firstly, as demonstrated in our empirical results, their antifungal efficacy is limited by the structural barriers of fungal cell walls, meaning they cannot serve as a universal cure for all sericultural diseases. Secondly, the antimicrobial activity and structural stability of the chitosan matrix depend heavily on environmental pH: its polycationic nature is most effective in slightly acidic to neutral environments, whereas highly alkaline conditions may induce nanoparticle aggregation or reduce the protonation of the functional amino groups, thereby weakening its electrostatic targeting capability. Furthermore, while we have established a safe in vivo dosage window for the silkworm host (*B. mori*), the long-term environmental fate and potential accumulation of silver nanoparticles in the broader sericultural ecosystem (e.g., mulberry field soils or groundwater) require rigorous, continuous monitoring. Therefore, the practical deployment of CS-Ag NPs must be carefully calibrated to take into account these environmental variables and the specific profiles of target pathogens to maximize efficacy while minimizing unintended ecological drawbacks.

## 5. Conclusions

In summary, rather than a simple superposition of safety and efficacy, the present study delineates a clear operational window for CS-Ag NPs in sericulture. By establishing a strict biosafety threshold that does not compromise larval development or silk production, it was demonstrated that prophylactic intervention within this safe dosage range can effectively extend the survival of hosts challenged by *S. marcescens* and directly inhibit BmNPV replication at the cellular level. Despite their limited efficacy against fungal pathogens with complex chitinous cell wall barriers, this inherent selectivity confers significant ecological advantages by minimizing potential risks to non-target organisms. The CS-Ag NPs developed in this study not only serve as a core intervention for the ecofriendly management of bacterial septicemia and viral nucleopolyhedrosis in sericulture but also provide critical theoretical frameworks and empirical evidence to support the future development of nanomaterial-based precision sericultural pharmaceuticals.

## Data Availability

The original contributions presented in this study are included in the article/supplementary material. Further inquiries can be directed to the corresponding author.

## Supplementary Materials

The following supporting information can be downloaded at: https://www.mdpi.com/article/10.3390/insects17040403/s1, Table S1: Primers used in the experiment.

## Abbreviations

The following abbreviations are used in this manuscript:

CCK	Cell Counting Kit
CS	Chitosan
CS-Ag NPs	Chitosan Silver Nanoparticles
MIC	Minimum Inhibitory Concentration
RT-qPCR	reverse transcription-quantitative polymerase chain reaction
UV	Ultraviolet–visible spectroscopy

## References

[1] Tatsuke T. (2024). Complete genome sequence of a nucleopolyhedrovirus isolated from *Bombyx mori* in Hakozaki, Japan, obtained using Oxford Nanopore Technologies sequencing. Microbiol. Resour. Announc..

[2] Guo D., Liu B.W., Cui M.X., Qian H.Y., Li G. (2025). Regulation of *Bombyx mori*–BmNPV protein interactions: Study strategies and molecular mechanisms. Viruses.

[3] Wang H.Y., Peng H., Li W.J., Cheng P., Gong M.Q. (2021). The toxins of *Beauveria bassiana* and the strategies to improve their virulence to insects. Front. Microbiol..

[4] Filippou C., Coutts R.H.A., Kotta Loizou I., El Kamand S., Papanicolaou A. (2025). Transcriptomic analysis reveals molecular mechanisms underpinning mycovirus-mediated hypervirulence in *Beauveria bassiana* infecting *Tenebrio molitor*. J. Fungi.

[5] Yin F., Xiao M.M., Berestetskiy A., Hu Q.B. (2021). The *Metarhizium anisopliae* Toxin, Destruxin A, Interacts with the SEC23A and TEME214 Proteins of *Bombyx mori*. J. Fungi.

[6] Syraji Y., Jeyaramraja P.R., Mada T., Gobikanila K. (2025). Comprehensive review of aflatoxin contamination, its occurrence, effects, management, and future perspectives. Discov. Food.

[7] Zhao P.F., Hong S., Li Y.K., Chen H.M., Gao H.C., Wang C.S. (2024). From phyllosphere to insect cuticles: Silkworms gather antifungal bacteria from mulberry leaves to battle fungal parasite attacks. Microbiome.

[8] Chi N.M., Nhung N.P., Van Loi V., Thuy P.T.T., Dell B. (2024). *Bacillus bombysepticus*, an entomopathogen in yellow-spined bamboo locust with biocontrol potential. Neotrop. Entomol..

[9] Bezuidenhout C.C., Molale Tom L.G., Kritzinger R.K., Olanrewaju O.S. (2023). Draft genome sequences of two *Bacillus bombysepticus* strains from drinking water. Microbiol. Resour. Announc..

[10] Qadir J., Bhat S.A., Rasheed S. (2025). Bacterial pathogens of silkworm: Impacts and management strategies. Int. J. Bio-Resour. Stress Manag..

[11] Ren C.J., Meng Y.C., Liu Y.Y., Wang Y., Wang H.J., Liu Y.T., Liu C.J., Fan X., Zhang S.X. (2025). Probiotic *Bacillus subtilis* enhances silkworm (*Bombyx mori*) growth performance and silk production via modulating gut microbiota and amino acid metabolism. Anim. Microbiome.

[12] Belyy A., Heilen P., Hagel P., Hofnagel O., Raunser S. (2023). Structure and activation mechanism of the Makes caterpillars floppy 1 toxin. Nat. Commun..

[13] Park J.W., Park S.K., Jeong C.Y., Kwon H.G., Lee J.H., Kang S.K., Kim S.-W., Kim S.-R. (2024). Microbial community changes in silkworms suspected of septicemia and identification of *Serratia* sp. Int. J. Mol. Sci..

[14] Mavilashaw V.P., Saranya M., Aruna N. (2025). Silkworm diseases and management: A review. Int. J. Adv. Biochem. Res..

[15] Boldeanu L., Boldeanu M.V., Novac M.B., Assani M.Z., Radu L. (2025). *Serratia marcescens*: A versatile opportunistic pathogen with emerging clinical and biotechnological significance. Int. J. Mol. Sci..

[16] Narzary P.R., Das A., Saikia M., Verma R., Sharma S., Kaman P.K., Boro R.C., Goswami S., Mahesh D.S., Linggi B. (2022). Recent trends in Seri-bioscience: Its prospects in modern sericulture. Pharma Innov..

[17] Tassoni L., Belluco S., Marzoli F., Contiero B., Cremasco S., Saviane A., Cappellozza S., Dalle Zotte A. (2024). Microbiological safety assessment of silkworm farms: A case study. Animal.

[18] Cappellozza S., Casartelli M., Sandrelli F., Saviane A., Tettamanti G. (2022). Silkworm and silk: Traditional and innovative applications. Insects.

[19] Strathdee S.A., Hatfull G.F., Mutalik V.K., Schooley R.T. (2023). Phage therapy: From biological mechanisms to future directions. Cell.

[20] Ashrith S., Pavithra M.R., Basangouda J., Shwetha G.V., Nandha K.R., Arun Kumar M.R., Suhas B.V. (2025). Advances in molecular breeding and CRISPR-Cas9-mediated genome editing in silkworms: Applications and future prospects. Int. J. Adv. Biochem. Res..

[21] Dukare Pradip G., Pavithra M., Thrilekha D., Ashrith S., Mala P.H., Bagde A.S. (2024). Application of nanotechnology in sericulture: A Review. J. Adv. Biol. Biotechnol..

[22] Deng B.Y., Dong Z.Q., Wu Q., Guo B.Y., Fang W.X., Hu C.W., Long J.Q., Chen P., Lu C., Pan M.H. (2022). Analysis of silver nanoparticles for the treatment and prevention of nucleopolyhedrovirus affecting *Bombyx mori*. Int. J. Mol. Sci..

[23] Montali A., Berini F., Gamberoni F., Armenia I., Saviane A., Cappellozza S., Gornati R., Bernardini G., Marinelli F., Tettamanti G. (2024). In vivo efficacy of a nanoconjugated glycopeptide antibiotic in silkworm larvae infected by *Staphylococcus aureus*. Insects.

[24] Bielska B., Miłowska K. (2025). Therapeutic potential of chitosan-based and related nanocomposite systems in wound management: A review. Int. J. Mol. Sci..

[25] Wahab S., Ali H.M., Khan M., Khan T., Krishnaraj C., Yun S.I. (2024). Green synthesis and antibacterial assessment of chitosan/silver nanocomposite conjugated with tobramycin against antibiotic resistant *Pseudomonas aeruginosa*. Arab. J. Chem..

[26] Karataş H., Eker F., Akdaşçi E., Bechelany M., Karav S. (2026). Silver nanoparticles in antibacterial research: Mechanisms, applications, and emerging perspectives. Int. J. Mol. Sci..

[27] Anees Ahmad S., Sachi Das S., Khatoon A., Tahir Ansari M., Afzal M., Saquib Hasnain M., Kumar Nayak A. (2020). Bactericidal activity of silver nanoparticles: A mechanistic review. Mater. Sci. Energy Technol..

[28] Korniienko V., Gogotsi O., Varava Y., Zandersone B., Deineka V., Husak Y., Diedkova K., Solodovnyk O., Kukurika V., Dukhnovskiy S. (2026). Critical assessment of intrinsic antibacterial properties and photothermal therapy potential of mxene nanosheets. ACS Appl. Nano Mater..

[29] Gong W.F., Sun Y.M., Tu T.T., Huang J.Y., Zhu C.Y., Zhang J.Q., Salah M., Zhao L.N., Xia X.S., Wang Y. (2024). Chitosan inhibits *Penicillium expansum* possibly by binding to DNA and triggering apoptosis. Int. J. Biol. Macromol..

[30] Žabka M., Pavela R. (2021). The dominance of chitosan hydrochloride over modern natural agents or basic substances in efficacy against *Phytophthora infestans*, and its safety for the non-target model species *Eisenia fetida*. Horticulturae.

[31] Luceri A., Francese R., Lembo D., Ferraris M., Balagna C. (2023). Silver nanoparticles: Review of antiviral properties, mechanism of action and applications. Microorganisms.

[32] Wang D.L., Yin C.Y., Bai Y.H., Zhou M.X., Wang N.D., Tong C.Y., Yang Y., Liu B. (2024). Chitosan-modified AgNPs efficiently inhibit swine coronavirus-induced host cell infections via targeting the spike protein. Biomolecules.

[33] Qiang M.D., Wu J.R., Zhang H.T., Zhan X.B. (2023). Ag/Cu-chitosan composite improves laundry hygiene and reduces silver emission in washing machines. Polymers.

[34] Lee S.W. (2023). Kaplan-Meier and Cox proportional hazards regression in survival analysis: Statistical standard and guideline of Life Cycle Committee. Life Cycle.

[35] Liu Z.Y., Li C., Yang W.Y., Wu Q., Xiao W.F., Zhu Y., Wei Q.Q., Dong Z.Q., Zhang G.Z., Lu C. (2024). The *Bombyx mori singed* gene is involved in the high-temperature resistance of silkworms. Insects.

[36] Zhang X.F., Liu Z.G., Shen W., Gurunathan S. (2016). Silver nanoparticles: Synthesis, characterization, properties, applications, and therapeutic approaches. Int. J. Mol. Sci..

[37] Confederat L.G., Tuchilus C.G., Dragan M., Sha’at M., Dragostin O.M. (2021). Preparation and antimicrobial activity of chitosan and its derivatives: A concise review. Molecules.

[38] Nasaj M., Chehelgerdi M., Asghari B., Ahmadieh-Yazdi A., Asgari M., Kabiri-Samani S., Sharifi E., Arabestani M. (2024). Factors influencing the antimicrobial mechanism of chitosan action and its derivatives: A review. Int. J. Biol. Macromol..

[39] Shi S.F., Shi W.R., Zhou B., Qiu S. (2024). Research and application of chitosan nanoparticles in orthopedic infections. Int. J. Nanomed..

[40] Alabbosh K.F., Elmetwalli A., Algehainy N.A., Altemani F.H. (2025). From bacterial extract to breakthrough therapy: *Pseudomonas fluorescens*-Enabled green synthesis of pH-responsive chitosan-silver hybrid nanoparticles for next-generation pulmonary drug delivery anti-MDR treatment. Pharmaceutics.

[41] Fan M.N., Si J.X., Xu X.G., Chen L.F., Chen J.P., Yang C., Zhu J.W., Wu L.H., Tian J., Chen X.Y. (2021). A versatile chitosan nanogel capable of generating AgNPs *in-situ* and long-acting slow-release of Ag^+^ for highly efficient antibacterial. Carbohydr. Polym..

[42] More P.R., Pandit S., Filippis A., Franci G., Mijakovic I., Galdiero M. (2023). Silver nanoparticles: Bactericidal and mechanistic approach against drug resistant pathogens. Microorganisms.

[43] Chopade P., Raghavendra C.G., S M.K., R.N. B. (2021). Assessment of diseases in *Bombyx mori* silkworm—A survey. Glob. Transit. Proc..

[44] Zhu X.Y., Xiao J., Li Y., Lei X.Y., Zhang H.R., Qian Z.Y., Sun C., Shao Y.Q. (2025). Sericulture mechanization poses new challenges for environmental disinfection—Evaluating the effects of three newly introduced disinfectants. AgriEngineering.

[45] Aflakian F., Mirzavi F., Aiyelabegan H.T., Soleimani A., Gholizadeh Navashenaq J., Karimi-Sani I., Rafati Zomorodi A., Vakili-Ghartavol R. (2023). Nanoparticles-based therapeutics for the management of bacterial infections: A special emphasis on FDA approved products and clinical trials. Eur. J. Pharm. Sci..

[46] Kallivalappil Puthalath A., Hazel S., Kottappara R., Srinivasan A., Vijayan B.K., Palantavida S. (2021). Synthesis and antibacterial activity of silver-copper nano-composites formed by microwave assisted chemical reduction. Mater. Today Proc..

[47] He Y.J., Chen J., Xu Z., Nie J.Q., Wang F.Y., Ma C., Wang C., Zhang L., Lu C.F. (2025). Silver functionalized chitosan composite hydrogel with sustained silver release and enhanced antibacterial properties promotes healing of infected wounds. Int. J. Biol. Macromol..

[48] Dube E., Okuthe G.E. (2025). Silver nanoparticle-based antimicrobial coatings: Sustainable strategies for microbial contamination control. Microbiol. Res..

[49] Gasti T., Dixit S., Hiremani V.D., Chougale R.B., Masti S.P., Vootla S.K., Mudigoudra B.S. (2022). Chitosan/pullulan based films incorporated with clove essential oil loaded chitosan-ZnO hybrid nanoparticles for active food packaging. Carbohydr. Polym..

[50] Yusof N.A.A., Zain N.M., Pauzi N. (2019). Synthesis of ZnO nanoparticles with chitosan as stabilizing agent and their antibacterial properties against Gram-positive and Gram-negative bacteria. Int. J. Biol. Macromol..

[51] Masood H.A., Qi Y.T., Zahid M.K., Li Z.T., Ahmad S., Lv J.M., Shahid M.S., Ali H.E., Ondrasek G., Qi X.J. (2024). Recent advances in nano-enabled immunomodulation for enhancing plant resilience against phytopathogens. Front. Plant Sci..

[52] Robinson J.R., Isikhuemhen O.S., Anike F.N. (2021). Fungal-metal interactions: A review of toxicity and homeostasis. J. Fungi.

