# Supplementary material for: Synergistic Antimicrobial and Antiviral Efficacy of Chitosan–Silver Nanocomposites Against Major Pathogens of Bombyx mori: In Vitro and In Vivo Evaluations
Source: Insects. 2026 Apr 8;17(4):403. doi: 10.3390/insects17040403 (PMC13115892; doi:10.3390/insects17040403)
Supplement: Supplementary file 1 [file insects-17-00403-s001.zip › insects-4171018-supplementary.pdf]

Supplementary Materials

Table S1. Primers used in the experiment

Primer	Sequences (5’-3’)
Bombyx mori-Ie-1-F	CGACTACAATTCCAACAGGTCGACTACAATTCCAACAGG
Bombyx mori-Ie-1-R	ATTTCAACGGCTTTACTTCATTTCAACGGCTTTACTTC
